# Clinical Significance of ABCG2/BCRP Quantified by Fluorescent Nanoparticles in Breast Cancer Patients Undergoing Neoadjuvant Chemotherapy

**DOI:** 10.3390/cancers15082365

**Published:** 2023-04-18

**Authors:** Hiroshi Tada, Kohsuke Gonda, Narufumi Kitamura, Takanori Ishida

**Affiliations:** 1 Division of Breast and Endocrine Surgical Oncology, Tohoku University Graduate School of Medicine, Sendai 980-8575, Miyagi, Japan; 2 Department of Medical Physics, Tohoku University Graduate School of Medicine, Sendai 980-8575, Miyagi, Japan

**Keywords:** breast cancer, breast cancer resistance protein, phosphor-integrated dots, chemotherapy resistance

## Abstract

**Simple Summary:**

Breast cancer resistance protein (BCRP) is a drug efflux pump associated with chemotherapy resistance. Effective quantitative analysis of BCRP expression in breast cancer tissue is essential for predicting breast cancer survival. Our laboratory developed nanoparticles suitable for quantitative immunohistochemical (IHC) analysis, called phosphor-integrated dots (PIDs). We examined the association between BCRP expression and prognosis among primary breast tumor sites and metastatic lymph nodes after neoadjuvant chemotherapy (NAC) in 37 breast cancer patients using IHC with PIDs at the single-particle level. The results show that overall survival was significantly worse in the high-BCRP-expression group (log-rank *p* = 0.0089). Quantitative BCRP expression analysis of primary tumors and breast cancer metastases could predict the prognosis of breast cancer patients after NAC. The IHC-PIDs were effective at the quantitative detection of the biomarker protein.

**Abstract:**

Breast cancer resistance protein (BCRP), also known as ATP-binding cassette transporter G2 (ABCG2), is associated with chemotherapy resistance. BCRP is also implicated in breast cancer stem cells, and is reported as a poor prognostic factor. However, the relationship of BCRP levels in breast cancer tissues with chemotherapy resistance and prognosis has not been clarified. We aimed to evaluate the correlation between BCRP expression and prognosis in breast cancer using immunohistochemistry with fluorescent phosphor-integrated dots (IHC-PIDs). A total of 37 breast cancer patients with residual cancer in the primary tumor and axillary lymph nodes were evaluated. BCRP levels in breast cancer tissue and metastatic lymph nodes were quantitatively detected after neoadjuvant chemotherapy (NAC). Among these 37 patients, 24 had corresponding core needle biopsies obtained before NAC. Biomarker assay with IHC-PIDs showed high accuracy for the quantitative assessment of BCRP with low expression. High BCRP expression in the primary tumor and metastatic lymph nodes after preoperative chemotherapy was associated with worse overall survival. In conclusion, high BCRP levels may be associated with poor prognosis in patients with breast cancer, having residual tumors within the primary tumor and lymph nodes after preoperative chemotherapy. These findings provide a basis for further appropriate adjuvant therapy in these patients.

## 1. Introduction

Neoadjuvant chemotherapy (NAC) is a common treatment modality for locally advanced breast cancer. It can enable surgery for inoperable breast cancers and improve breast conservation rates for operable but large breast tumors, and is critical to identify factors predictive of subsequent clinical outcomes after NAC [1]. The pathologic complete response (pCR) in primary breast cancer has been reported to be associated with higher rates of disease-free survival (DFS) and overall survival (OS) in patients treated with NAC [2,3]. However, pCR is not a useful prognostic factor, especially for hormone-receptor-positive breast cancers [4]. A recent systematic review and meta-analysis showed that both DFS and OS were insufficient surrogate endpoints for clinical trials [5]. In addition, recurrence after a long period (≥10 years) is also a problem in hormone-receptor-positive breast cancers, and thus, biomarkers to predict long-term prognosis are needed.

ATP-binding cassette (ABC) subfamily G isoform 2 protein (ABCG2), also known as breast cancer resistance protein (BCRP), is a drug efflux pump and an important member of the ABC transporter superfamily [6,7]. Several cytotoxic drugs, including those used to treat breast cancer, are substrates for ABCG2, and resistance to these drugs is thought to be the result of drug efflux by ABCG2 [8,9]. ABCG2 has also been implicated in the cancer stem cell (CSC) phenotype. CSCs have been hypothesized to play a role in tumorigenesis, resistance, recurrence, metastasis, and tumor heterogeneity [10,11,12].

Our laboratory recently developed nanoparticles suitable for the quantitative immunohistochemical (IHC) method called phosphor-integrated dots (PIDs). Using PID-based IHC (IHC-PID), many biomarker proteins can be visualized and quantitatively analyzed at the single-particle level in paraffine-embedded, formalin-fixed tumor samples [13,14]. The IHC-PID method is a versatile optical technique that can accurately estimate protein expression independent of the optical system. IHC-PID can quantitatively identify single PIDs that recognize target molecules in fluorescent immunostaining using versatile optical microscopy. The number of PID particles is an absolute value, and does not vary across systems [13]. This study aimed to examine the correlation of survival and long-term follow-up with BCRP expression in primary and metastatic lesions following NAC over a long-term follow-up period. Toward this goal, we examined the differences in BCRP expression among primary breast tumor sites and metastatic lymph nodes after NAC in breast cancer patients using fluorescent IHC-PIDs. We hypothesized that quantitative BCRP expression analysis of primary tumors and metastases of breast cancer could predict the prognosis of breast cancer patients after NAC.

## 2. Materials and Methods

### 2.1. Study Design and Patients

This retrospective study was approved by the Ethics Committee of the Graduate School of Medicine at Tohoku University (No. 2021-1-353). The need for informed consent was waived owing to the retrospective nature of the study, and the option to opt out was used to obtain consent for this study on our homepage.

Thirty-seven Japanese patients diagnosed with invasive ductal carcinoma of the breast who underwent a mastectomy and lymph node dissection at Tohoku University Hospital (Sendai, Japan) between 2004 and 2010 were included. All patients had undergone NAC, and were pathologically diagnosed with non-pathological complete response or progressive disease and lymph node metastasis postoperatively. The patients were followed up within a median of 10.1 years (range, 1.1–18.2 years). Among the 37 patients, 28 patients had core needle biopsy specimens taken before NAC.

In this study, breast cancer subtypes were classified by IHC analysis of the estrogen receptor (ER), progesterone receptor (PgR), human epidermal growth factor receptor type 2 (HER2), and the Ki-67 index. The following subtypes were defined: luminal-type (ER+ and/or PgR+), HER2-type (ER− and PgR−, and HER2+), and triple-negative (TN; ER− and PgR−, and HER2−). The luminal type was further subdivided into types A (HER2− and Ki-67 < 20.0%) and B (HER2+ and/or Ki-67 ≥ 20%).

The clinicodemographic patient characteristics are summarized in Table 1.

Following the classification published by the Japanese Breast Cancer Society, the pathological response was determined by combining the degree of degeneration of cancer cells (mild change, significant change) and the area of degenerated cancer cells, classified into five grades: 0 (no response), 1a (mild response), 1b (moderate response), 2 (marked response), and grade 3 (complete response) (version 2007) [15]. No patients had grade 0 or grade 3 pathological responses, and all patients included had grade 1a, 1b, or 2 diseases. No patients received preoperative endocrine therapy. There was no significant correlation between the type of NAC regimen (with or without taxane) and clinical outcomes.

The primary endpoints were DFS and OS according to BCRP levels in the primary tumor of core needle biopsy and surgical specimens and metastatic lymph nodes after NAC. The secondary endpoint was to determine the relationship between clinicopathologic characteristics and differential BCRP expression after NAC.

### 2.2. Fluorescence Immunohistochemistry Staining

Paraffin sections of tumor samples were heated for 15 min at 65 °C, deparaffinized in xylene, and hydrated using an alcohol gradient with distilled water. Antigen retrieval was performed in Tris EDTA buffer (pH 9) for 40 min at 95 °C. Cells cultured in 35 mm glass-bottom dishes were fixed with 4% paraformaldehyde for 15 min at room temperature (RT, 20–25 °C). After the samples were washed in phosphate-buffered saline, endogenous peroxidases and nonspecific binding sites were sequentially blocked by incubation with an endogenous biotin blocking kit (Ventana, Tokyo, Japan) for 10 min and with 10% goat serum (Funakoshi, Tokyo, Japan) in phosphate-buffered saline for 1 h at RT. Cells were immunostained with a primary anti-BCRP antibody (mouse monoclonal, clone BXP-21 at 1:200; Millipore Sigma, Burlington, MA, USA) overnight at 4 °C. After washing, the samples were incubated for 30 min with a biotinylated goat anti-rabbit IgG secondary antibody (Southern Biotech, Birmingham, AL, USA) diluted in a 1:50 ratio with a Dako antibody diluent (Agilent Technologies, Santa Clara, CA, USA). Samples were then incubated with 0.02-nM PIDs for 2 h, and then with DAPI for 10 min at RT. Furthermore, nuclei were stained with hematoxylin and mounted using a Malinol mounting medium (Muto Pure Chemicals Co., Ltd., Tokyo, Japan). After the final wash, the samples were mounted in ProlongTM gold antifade reagent (Invitrogen, Waltham, MA, USA).

### 2.3. IHC-PIDs

**Figure 1:**
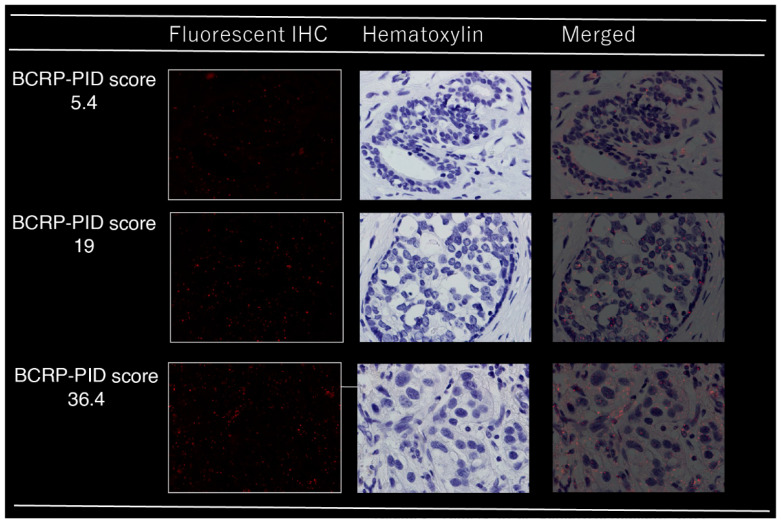
Immunohistochemical analysis of BCRP in breast cancer tissues using phosphor-integrated dot (PID) staining; red spots on tumor cells indicate PID particles.

The fluorescence signals of PIDs were observed using fluorescence microscopy (BX53; Olympus Corp., Tokyo, Japan) with a UPLSAPO 40 × 2 (Olympus) objective lens and charge-coupled device camera (DP73; Olympus) in five microscopic fields (with ~1000 cells investigated in each sample). The PID score (PIDs per cell) was thereafter determined (Figure 1) [13,14].

### 2.4. Statistical Analysis

Significant differences in clinicopathologic characteristics between groups were assessed using either the χ^2^ test or *t*-test. Unpaired *t*-tests and one-way analysis of variance were used to analyze differences in BCRP expression among OS and DFS events. OS and DFS curves were generated using the Kaplan–Meier method and compared using the log-rank test. Independent prognostic factors were identified using backward stepwise multivariate Cox regression analyses with covariates that were significantly associated with OS in the univariate analysis. Hazard ratios and their 95% confidence intervals (CIs) were calculated for each factor. All statistical analyses were performed using JMP Pro software version 16.2.0 (SAS Institute, Cary, NC, USA). All tests were two-tailed, and *p* ≤ 0.05 was considered significant.

## 3. Results

### 3.1. Quantitative Immunostaining of BCRP

Our laboratory initially developed the IHC-PIDs and an image processing method to calculate human epidermal growth factor receptor 2 (HER2) expression with high accuracy using the PID score [13]. Given that BCRP, similar to HER2, is present on the plasma membrane, we performed a quantitative analysis of BCRP using the same method. The fluorescent immunostaining images and PID scores are shown in Figure 1. Concerning the association of clinicodemographic factors with BCRP PID scores in core needle biopsy (CNB) samples obtained before NAC, for both primary tumor and lymph node samples, the only significant correlation was with age (Table S1). There were no significant differences in the mean BCRP-PID between CNB samples and matched samples of BCRP expression in primary breast cancer tissue (PT) after NAC (15.7 vs. 15.2; mean difference, 0.56; standard error, 1.57; *p* = 0.72). BCRP expression was significantly lower in lymph node (LN) samples than in CNB samples, and in LNs after NAC (mean score, 11.3 vs. 15.2; mean difference, −3.91; standard error, 1.10; *p* = 0.0014).

### 3.2. Predictive Capability of BCRP IHC-PIDs for NAC Response

**Figure 2:**
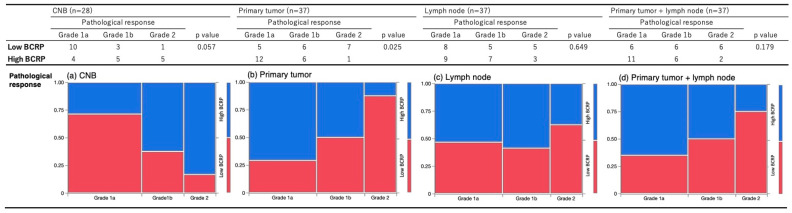
Association between BCRP expression binarized by the median and pathological response in (**a**) CNB, (**b**) primary tumor, (**c**) metastatic lymph node, and (**d**) primary tumor and metastatic lymph node samples; blue, low BCRP; red, high BCRP.

Pathological responses differed according to BCRP expression (high vs. low) in CNB, PT, and metastatic lymph node samples (Figure 2a–c). In PT samples, the higher the BCRP expression, the worse the response to NAC (*p* = 0.025, Figure 2b). Similarly, in PT + LN samples, residual tumors that did not respond to NAC also showed higher BCRP expression, although the difference was not significant (*p* = 0.179, Figure 2d). In contrast, in CNB samples before NAC, BCRP tended to be lower in cases that responded poorly to NAC (*p* = 0.057, Figure 2a). There was no significant difference between BCRP and pathological response in lymph nodes (*p* = 0.649, Figure 2c).

### 3.3. Correlation between Biological Factors and Survival Events

We compared the incidence of DFS and OS events for each clinicodemographic factor (age, histologic grade, lymph node count, pStage, pathologic response) and the extent of BCRP expression (Table 2). For clinicodemographic factors, OS events significantly differed according to the Ki-67 index (*p* = 0.009), but not according to age, histological grade, number of lymph node metastases, pStage, or pathologic response. Meanwhile, there was no significant difference in DFS and OS events according to BCRP levels in CNB, PT, metastatic axillary LN, and PT + LN samples (*p* = 0.445, *p* = 0.419, *p* = 0.603, and *p* = 0.886 for DFS events and *p* = 0.430, *p* = 0.385, *p* = 0.842, and *p* = 0.124 for OS events, respectively).

**Figure 3:**
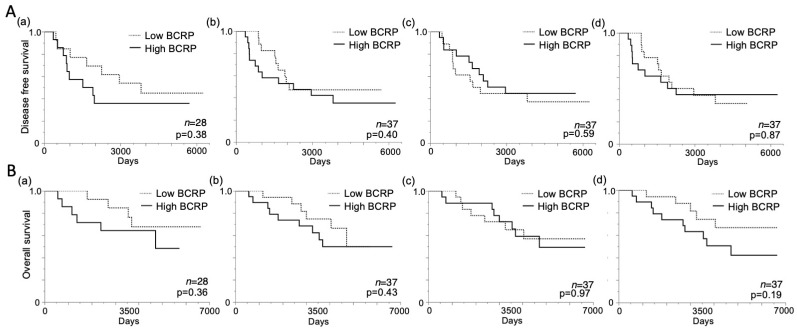
Kaplan–Meier disease-free survival (DFS) and overall survival (OS) curves according to BCRP expression. (**A**) DFS according to median BCRP expression in (**a**) core needle biopsy (CNB), (**b**) primary tumor, (**c**) metastatic lymph node, and (**d**) primary tumor and metastatic lymph node samples. (**B**) OS according to the median BCRP expression in (**a**) CNB, (**b**) primary tumor, (**c**) metastatic lymph node, and (**d**) primary tumor and metastatic lymph node samples; *p*-values were determined using the log-rank test.

We then analyzed correlations between continuous variables and BCRP expression in CNB, PT, LN, and PT + LN samples with respect to DFS and OS events (Table 3). The results show significant differences in OS events in CNB samples (*p* = 0.07), but not in other samples. BCRP expression in CNB, PT, LN, and PT + LN samples was dichotomized into high and low according to the median. Kaplan–Meier curves for OS and DFS were generated and compared between the two groups (Figure 3A,B). In CNB samples, both DFS and OS tended to be worse in the high-BCRP group (log-rank *p* = 0.38, Figure 3A(a); log-rank *p* = 0.36, Figure 3B(a)). Although prognosis was not significantly different according to BCPR expression in PT and LN samples (Figure 3A(b,c),B(b,c)), there was a significant trend toward worse OS in PT + LN samples with high BCRP expression (log-rank *p* = 0.19; Figure 3B(d)).

**Figure 4:**
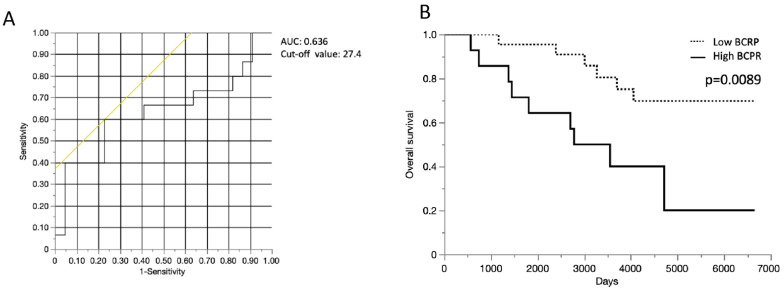
Kaplan–Meier overall survival (OS) curves according to BCRP expression cut-off in primary tumors and metastatic lymph nodes. (**A**) Receiver operating characteristic analysis of the usefulness of BCRP expression in primary tumor and metastatic lymph node samples for predicting death and survival (*n* = 37 patients). (**B**) OS curves according to BCRP expression cut-off in primary tumors and metastatic lymph nodes. AUC, area under the curve.

To investigate the correlation between OS and BCRP expression in PT + LN samples, we evaluated PT + LN PID scores according to cut-off values determined using receiver-operating characteristic curves based on the highest sum of sensitivity and specificity for BC recurrence within 10 years (Figure 4A). The PT + LN BCRP score displayed high discriminating power for differentiating between non-survivors and survivors, with an area under the curve of 0.63. The optimal PT + LN BCRP cut-off score for predicting survival was 27.4, with a sensitivity of 60.6% and a specificity of 77.3%. BCRP expression in PT + LN samples was dichotomized into high and low groups at this cut-off value, and OS was compared between these groups. The results show that OS was significantly worse in the high-BCRP-expression group (log-rank *p* = 0.0089; Figure 4B).

In univariate analysis, high PT + LN BCPR scores (≥27.4) and high Ki-67 values (≧20) were significantly associated with worse OS (*p* = 0.03 and *p* = 0.002, respectively; Table 4). In multivariate analysis, although there was no independent prognostic factor for OS, Ki-67 (hazard ratio: 3.21, 95% CI: 0.93–11.01, *p* = 0.06) and PT + LN BCRP expression (hazard ratio, 2.67; 95% CI, 0.81–8.79; *p* = 0.11; Table 4) were close to significant prognostic factors for OS.

## 4. Discussion

In this study, we examined the association between BCRP expression and prognosis, and found that high BCRP expression in surviving cancer cells in PT and LN samples after NAC was significantly associated with poor OS. In recent years, microarray-based methods have been used to predict the prognosis of patients with breast cancer [16,17]. However, prognostic prediction using a single biomarker in the primary tumor tissue is essential because it improves the ease of prediction and presents a potential target substance for targeted therapy. Marginal cases, excluding those with poor (grade 0) and sound (grade 3) pathological responses, were included in this study because surgical and lymph node specimens were not available for cases of grades 0 and 3. It was expected that pre-NAC CNB samples with high BCRP expression would be less responsive to NAC, perhaps because of the inclusion of such marginal cases in this study. The prognosis of patients with grade 1–2 pathological responses is unclear, but one study reported that patients with grade 2 responses had a better prognosis [18]. The long observation period of the current study (10.1 years) allowed for adequate survival analysis. The results show a significant difference in OS between high- and low-BCRP-expression cases in the PT + LN group. There was a trend toward shorter post-relapse survival in the PT + LN high-BCRP-expression group than in the PT + LN low-BCRP-expression group. This suggests that patients who had more BCRP-rich cells after NAC may have retained high BCRP expression after relapse, and may have been resistant to various chemotherapy regimens after relapse. Therefore, increasing the intensity of adjuvant therapy or using molecular therapies targeting BCRP for patients with high BCRP levels after NAC may be desirable. It could also lead to the assembly of new response-guided plus biomarker-guided treatment strategies.

To our knowledge, this study is the first to analyze BCRP expression before and after chemotherapy in patients with breast cancer who underwent surgery. A comparison of BCRP levels in paired specimens of CNB and LN samples after NAC showed significantly lower levels in LNs. This difference could be due to the heterogeneity of breast cancer tumors [19] and the site dependence of CSCs [20]. Breast cancer forms heterogeneous tumors with many subclones, and intratumoral heterogeneity is well known in primary and metastatic tumors [21]. Our previous study found that the LN metastasis subtype is a good prognostic factor in patients undergoing NAC [22].

Our findings show that the Ki-67 index, which is a cell proliferation marker, correlated with prognosis but not with BCRP expression. Furthermore, BCRP levels were not correlated with histological grade or the number of lymph node metastases, which were correlated with prognosis after NAC. These findings support BCRP expression as an independent predictor of prognosis. Higher BCRP expression in CNB samples was associated with a poorer response to NAC, and thus, it could be a marker of NAC resistance. This result can be explained biologically [23,24]. In addition, BCRP expression was higher after NAC, suggesting that many of the surviving tumor cells were chemotherapy-resistant. The association between BCRP levels and chemotherapy resistance was similar to a previous report [25].

In our previous study, we found that the overall expression of BCRP was lower than that of HER2 [13], indicating that the former is expressed at relatively low levels. Conventional immunostaining of BCRP was not performed in the present study because of the low expression of BCRP and the difficulty of quantifying the protein biomarker with high sensitivity using conventional IHC. IHC-PID can quantify even low-expression proteins, and it has the advantage of quantitatively analyzing the expression of individual cell biomarkers even in specimens with a low residual tumor volume after NAC. New therapeutic agents for breast cancer that exhibit drug resistance have recently emerged, such as Sacituzumab govitecan-hziy [26], trastuzumab-deruxtecan [27], and other antibody–drug conjugates. However, there is no biomarker for predicting the therapeutic efficacy of these drugs. The evaluation of BCRP by IHC-PID is worth considering as one of the biomarkers for using these agents in non-CR cases. In addition, if molecular targeted drugs against BCRP are to be established as effective in the future, BCRP quantification by PIDs may be helpful as a complementary diagnostic modality for determining the usefulness of new drugs targeting BCRP.

This study has some limitations that should be considered when interpreting the results. First, the small sample size reduced the ability to use ABCG2/BCRP expression as a prognostic and treatment resistance factor. In addition, although the study included patients diagnosed between 2004 and 2010 to obtain a sufficient observation period, only a small number of patients received preoperative chemotherapy in our institution. In addition, the IHC-PID analysis is currently research-based, and thus, it was not possible to study a large number of cases. However, we believe that bias is minimal because the study was conducted at a single institution with consecutive patients for whom tissue specimens were available. Second, this was a retrospective observational study. Third, the IHC-PID method used in this study is a PID-nanoparticle-based method. This IHC-PID method can only be performed in a limited number of facilities. However, it is an excellent method for the quantification of protein biomarkers. It has been used to quantify colony-stimulating factor 1 receptor-expressing tumor-associated macrophages in lung cancer [28] and programmed death ligand 1 expression in pancreatic ductal carcinoma [29]. The IHC-PID method is promising for formalin-fixed, paraffin-embedded sections, especially for protein biomarkers with low expression, and when accurate quantification is required. Large-scale prospective studies are needed to validate our findings.

## 5. Conclusions

High BCRP expression in residual tumor cells may be indicated resistance to NAC, and can therefore be used to predict the long-term outcomes of patients with breast cancer undergoing NAC. Further stratification of patients after surgery may extend treatment options.

## Figures and Tables

**Table 1:** Clinicopathological patient characteristics.

Patient Characteristics		N (*n* = 37)	%
Age (years)	<50	15	40.5
(Median: 54 (31–76))	≥50	22	59.5
pTNM	Stage I	0	0.0
	Stage II	22	59.5
	Stage III	15	40.5
	Stage IV	0	0.0
Number of	1–3 (pN1)	17	45.9
metastatic lymph nodes	4–9 (pN2)	13	35.1
	≥10 (pN3)	7	18.9
Ki67 LI (%)	<20	28	75.7
	≥20	9	24.3
Histological Grade	1	4	10.8
(Primary tumor)	2	20	54.1
	3	4	10.8
Subtype	Luminal A	19	51.4
(Primary tumor)	Luminal B—HER2−	11	29.7
	Luminal B—HER2+	5	13.5
	HER2	0	0.0
	Triple negative	2	5.4
Regimen	Anthracycline	5	13.5
	Anthracycline + Taxane	27	73.0
	Anthracycline + Taxane + Trastuzumab	5	13.5
Pathological response	0	0	0.0
	1a	17	45.9
	1b	12	32.4
	2	8	21.6
	3	0	0.0

**Table 2:** Correlations between biological factors and recurrence or survival.

		*n* (%)					*n* (%)				
Characteristics		No Recurrence		Recurrence		*p*-Value	Survival		Death		*p*-Value
		(*n* = 16)		(*n* = 21)			(*n* = 22)		(*n* = 15)		
Age (years)	<50	6	(16.2)	9	(24.3)	0.742	10	(27.0)	5	(13.5)	0.461
	≥50	10	(27.0)	12	(32.4)		12	(32.4)	10	(27.0)	
Histological grade	Grade 1–2	14	(37.8)	16	(43.2)	0.384	20	(54.1)	10	(27.0)	0.065
(Primary tumor)	Grade 3	2	(5.4)	5	(13.5)		2	(5.4)	5	(13.5)	
Ki-67	<20	14	(37.8)	14	(37.8)	0.143	20	(54.1)	8	(21.6)	0.009
(Primary tumor)	≥20	2	(5.4)	7	(18.9)		2	(5.4)	7	(18.9)	
No. of LN metastasis	<4	9	(24.3)	8	(21.6)	0.272	12	(32.4)	5	(13.5)	0.204
	≥4	7	(18.9)	13	(35.1)		10	(27.0)	10	(27.0)	
pStage	2	8	(21.6)	7	(18.9)	0.306	10	(27.0)	5	(13.5)	0.461
	3	8	(21.6)	14	(37.8)		12	(32.4)	10	(27.0)	
Pathological response	1a	9	(24.3)	8	(21.6)	0.417	10	(27.0)	7	(18.9)	0.756
	1b	5	(13.5)	7	(18.9)		8	(21.6)	4	(10.8)	
	2	2	(5.4)	6	(16.2)		4	(10.8)	4	(10.8)	
CNB	BCRP low	7	(18.9)	7	(18.9)	0.445	10	(27.0)	4	(10.8)	0.430
	BCRP high	5	(13.5)	9	(24.3)		8	(21.6)	6	(16.2)	
Primary tumor	BCRP low	9	(24.3)	9	(24.3)	0.419	12	(32.4)	6	(16.2)	0.385
	BCRP high	7	(18.9)	12	(32.4)		10	(27.0)	9	(24.3)	
Lymph node metastasis	BCRP low	7	(18.9)	11	(29.7)	0.603	11	(29.7)	7	(18.9)	0.842
	BCRP high	9	(24.3)	10	(27.0)		11	(29.7)	8	(21.6)	
Primary tumor +	BCRP low	8	(21.6)	10	(27.0)	0.886	13	(35.1)	5	(13.5)	0.124
Lymph node metastasis	BCRP high	8	(21.6)	11	(29.7)		9	(24.3)	10	(27.0)	

Pathological response 1a, mild; 1b, moderate; 2, marked.

**Table 3:** Correlations between BCRP-PID score and recurrence or survival.

		*n*	BCRP-PID Score (Mean ± S.E.)	95% CI	*p*-Value *
CNB (*n* = 28)	No Recurrence	12	14.3 ± 1.2	11.9–16.6	0.30
	Recurrence	16	15.9 ± 1.0	13.8–17.9	
PT (*n* = 37)	No recurrence	16	13.7 ± 1.8	1.8–10.1	0.61
	Recurrence	21	14.9 ± 1.5	1.5–11.8	
LN (*n* = 37)	No recurrence	16	10.3 ± 1.2	7.8–12.8	0.61
	Recurrence	21	11.1 ± 1.1	9.0–13.3	
PT + LN (*n* = 37)	No recurrence	16	24.0 ± 2.0	19.9–28.1	0.45
	Recurrence	21	26.0 ± 1.8	22.5–29.6	
CNB (*n* = 28)	Survival	18	14.2 ± 0.9	12.3–16.0	0.07
	Death	10	17.0 ± 1.2	14.5–19.5	
PT (*n* = 37)	Survival	22	13.4 ± 1.5	10.4–16.5	0.32
	Death	15	15.8 ± 1.8	12.1–19.4	
LN (*n* = 37)	Survival	22	10.3 ± 1.0	8.2–12.4	0.45
	Death	15	11.5 ± 1.2	9.0–14.1	
PT + LN (*n* = 37)	Survival	22	23.7 ± 1.7	20.3–27.1	0.19
	Death	15	27.3 ± 2.1	23.1–31.5	

* Mann–Whitney U-test; CNB, core needle biopsy; PT, primary tumor; LN, metastatic lymph node.

**Table 4:** Univariate and multivariate analyses of influencing factors of overall survival (*n* = 37).

	Univariate			Multivariate		
	Hazard Ratio	95% CI	*p*-Value	Hazard Ratio	95% CI	*p*-Value
Histological grade (PT)						
3/1–2	2.90	0.98–8.54	0.05	2.20	0.51–7.25	0.19
Ki67 (PT)						
≥20/<20	5.19	1.82–14.80	0.002	3.21	0.93–11.01	0.06
Lymph node metastasis						
pN2 + pN3/pN1	1.74	0.59–5.14	0.32			
pStage						
3/2	1.74	0.58–5.22	0.32			
Pathological response						
1/2	0.70	0.22–2.22	0.55			
BCRP (PT + LN, by cut-off)						
High/low	3.78	1.32–10.80	0.03	2.67	0.81–8.79	0.11

## Data Availability

Not applicable.

## Supplementary Materials

The following supporting information can be downloaded at https://www.mdpi.com/article/10.3390/cancers15082365/s1. Table S1. Association between BCRP expression binarized by median and clinicopathological factors. CNB, core needle biopsy; PT, primary tumor; LN, metastatic lymph node.
