# Supplementary material for: Clinical Significance of ABCG2/BCRP Quantified by Fluorescent Nanoparticles in Breast Cancer Patients Undergoing Neoadjuvant Chemotherapy
Source: Cancers (Basel). 2023 Apr 18;15(8):2365. doi: 10.3390/cancers15082365 (PMC10136557; doi:10.3390/cancers15082365)
Supplement: Supplementary file 1 [file cancers-15-02365-s001.zip › cancers-2251358-supplementary.pdf]

Supplementary Materials

Table S1. Association between BCRP expression binarized by median and clinicopathological factors

		BCRP PID score (CNB)			BCRP PID score (PT)			BCRP PID score (LN)		
		Low	High	p value	Low	High	p value	Low	High	p value
Age, years	<50	9	4	0.06	5	10	0.12	4	11	0.03
	≥50	5	10		13	9		14	8	
Histological grade (PT)	1	2	2	0.54	2	2	0.48	2	2	0.94
	2	11	9		14	12		13	13	
	3	1	3		2	5		3	4	
Number of metastatic lympn nodes	1-3 (pN1)	8	6	0.47	6	11	0.24	9	8	0.19
	4-9 (pN2)	3	6		7	6		4	9	
	≥10 (pN3)	3	2		5	2		5	2	
pStage	2a	7	2	0.23	5	5	0.64	3	7	0.39
	2b	1	2		1	4		4	1	
	3a	2	5		6	4		4	6	
	3b	1	3		2	3		3	2	
	3c	3	2		4	3		4	3	

CNB, core needle biopsy; PT, primary tumor; LN, metastatic lymph node.
